# Supplementary material for: Motus Vita Est: Fruit Flies Need to Be More Active and Sleep Less to Adapt to Either a Longer or Harder Life
Source: Clocks Sleep. 2023 Mar 3;5(1):98–115. doi: 10.3390/clockssleep5010011 (PMC10047790; doi:10.3390/clockssleep5010011)
Supplement: Supplementary file 1 [file clockssleep-05-00011-s001.zip › clockssleep-2113644-supplementary.pdf]

Supplementary

to

"Motus Vita Est: Fruit Flies Need to be More Active and Sleep Less to Adapt to Either a Longer or Harder Life"

Introduction to Supplementary

We also tested whether the difference in locomotor activity and sleep between four strains might be related to the difference in some of metabolic indicators (see the main text), and whether the difference between strains in some of life history traits including survival rate and fecundity persisted after their arrival in the Novosibirsk (since September 2021). The methods testing survival rate and fecundity, tables and figures with results of this testing are included in this Supplementary.

Methods in Brief

Testing Longevity and Fecundity

In order to test the difference in survival time, 20 flies were kept in each of 40 tubes at room temperature and natural photoperiod. Males and females from four strains were compared when living on either standard or starch food substrate (Table S1 and Figure S1). The food vials were replaced, at least, twice a week after removing and counting dead flies. In total, 800 flies (200 per each strain) were counted in this analysis of survival time.

Fecundity was tested after rearing flies either on the food substrates used for their selection in the Moscow university or on the standard food substrate used in the Novosibirsk institute in the previous studies of locomotor activity and sleep. Male and female offspring of 7 groups consisting of 5 males and 5 females from each of 4 strains were compared at the Moscow food substrates, and male and female offspring of 8 such groups were compared on the standard

food substrate (in total, 70 and 80 parent flies, respectively). Starting from age 5 days, flies have been kept together in such 10-fly groups for one day. After their removal, the numbers of male and female offspring were calculated, three and two times with 2-day intervals, respectively (Table S2).

Statistical Analysis

To test significance of difference between strains in survival time, the Kaplan–Meier estimator was used, and the log-rank (Mantel–Cox) test was applied for the comparisons of the Kaplan–Meier curves of four strains (Table S1).

Chi-square test, χ_2^2, was used for the comparisons of fecundity of strains (Table S2).

Results in Brief

Data on survival rate (Table S1 and Figure S1) confirmed that the difference between flies from the control strain and flies from three selected strains persisted over generations. For instance, artificial selection of flies for late reproduction yielded flies with longer than the control lifespan, while shorter lifespans were confirmed for flies from two strains adapted to a harder life on the adverse food substrates (Table S1 and Figure S1). The shortest lifespan was found in flies from the starch strain (Table S1 and Figure S1).

Moreover, selection on these two adverse food substrates also resulted in significant reductions of such direct measure of fitness as the number of offspring (Table S2).

Supplementary Tables

Table S1. Pairwise comparisons of survival time.

	Food substrate	Standard				Starch			
		Mean		Median		Mean		Median	
	Strain	Mean	SEM	Median	SEM	Mean	SEM	Median	SEM
Survival time in mean and median days									
Total	Control	55.81	1.31	60	0.97	40.30	1.00	43	0.54
	Long-lived	56.17	1.77	67	2.10	43.03	1.03	50	0.16
	Salt	43.29	1.32	45	2.02	38.66	0.66	38	0.68
	Starch	32.62	1.26	35	2.21	25.36	1.09	15	0.36
Male	Control	49.63	1.92	52	1.50	43.06	1.48	50	0.36
	Long-lived	50.08	2.59	59	5.00	41.84	1.59	50	0.24
	Salt	35.89	1.73	35	2.50	39.97	1.10	42	1.42
	Starch	26.15	1.55	24	3.65	27.68	1.56	15	1.82
Female	Control	61.98	1.55	67	2.03	37.54	1.29	42	0.86
	Long-lived	62.26	2.27	74	1.28	44.22	1.31	50	0.39
	Salt	50.68	1.71	59	2.68	37.35	0.72	35	0.54
	Starch	39.09	1.77	40	1.38	23.03	1.50	15	0.51
Pairwise comparison									
Sex					Male				Male
	Strain	Control	Long-lived	Salt	Starch	Control	Long-lived	Salt	Starch
	Control		9.75**	31.04***	88.73***		0.45	16.80***	41.67***
	Long-lived	14.02***		37.91***	69.84***	31.41***		15.66***	37.85***
	Salt	41.47***	60.38***		17.52***	9.30**	67.32***		16.05***
	Starch	72.47***	75.54***	16.32***		30.76***	88.83***	18.07***	
Sex		Female				Female			

Notes. Standard food substrate: the same standard diet as used for the flies in Table 1; Starch food substrate: The same diet that was used for selection of Starch strain; Mean and Median: Averaged and median survival time, days; SEM: Standard Error of these Mean and Median; $^{**}p < 0.01$ and $^{***}p < 0.001$ were obtained using Log Rank (Mantel-Cox) test in *post hoc* pairwise comparisons of four strains on survival time (the symbols are placed behind the estimate of χ^2). See also notes to Table 1. These results are illustrated in Figure S1.

Table S2. Pairwise comparisons of fecundity of the control strain with each of other three strains.

Food substrate	Number of offspring						Chi-squire test for total			
	Moscow			Standard			Moscow		Standard	
Strain	Total	Male	Female	Total	Male	Female	χ_2^2	p	χ_2^2	p
Control	574	271	303	146	76	70	Compared to Control			
Long-lived	578	286	292	114	57	57	0.014	0.906	3.938	0.047
Salt	219	104	115	102	58	44	158.9	<0.001	7.806	0.005
Starch	161	65	96	108	57	51	232.1	<0.001	5.685	0.017

Notes. Moscow: The same food substrates as used for selection of the strains in Moscow; Standard: the same standard food substrate as used for the flies in Table 1; χ_2^2: testing whether the number of offspring produced by each of three selected strains differ from that in the control strain.

Supplementary Figure

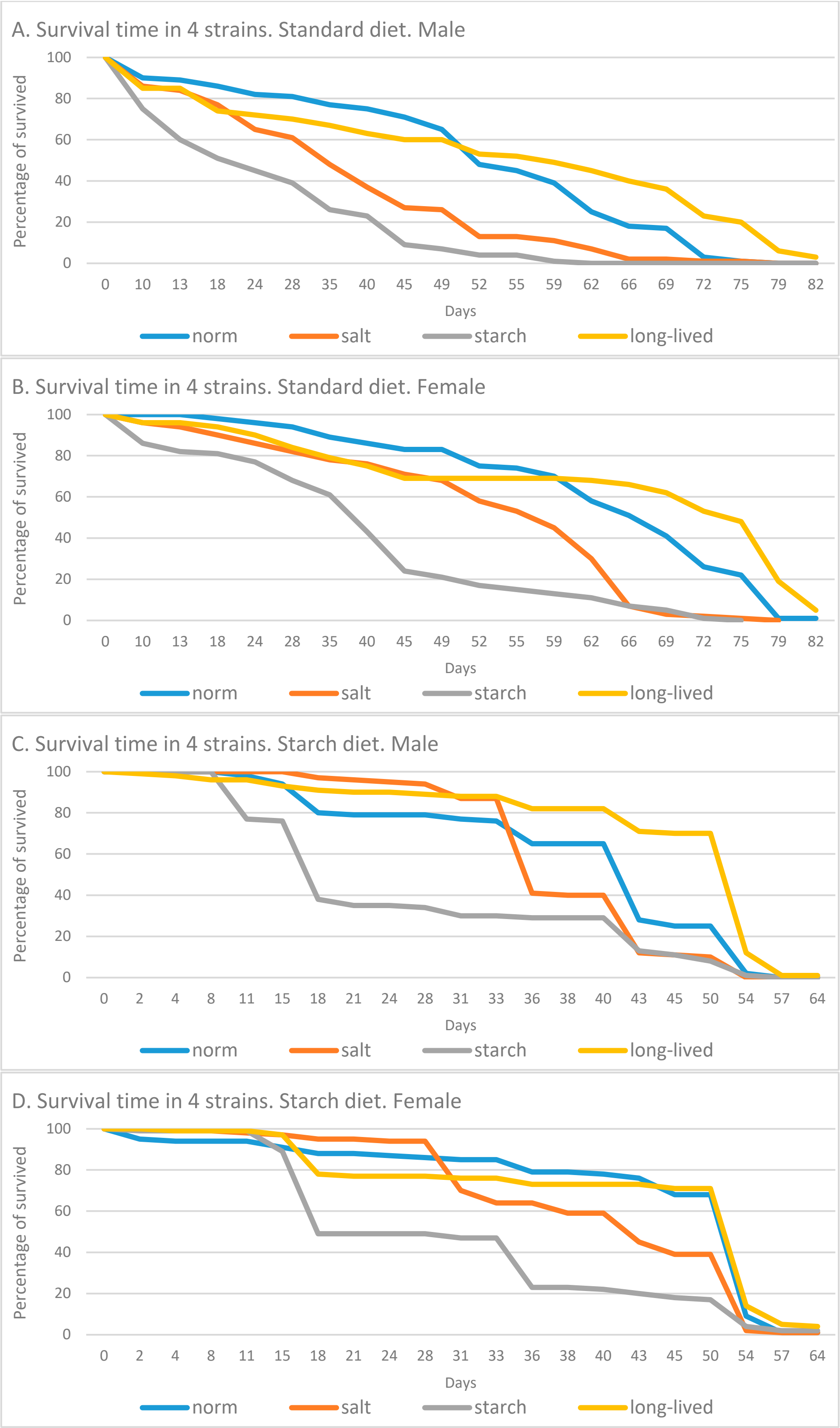

Figure S1
